# Analysis of the short-term efficacy of 2 versus 3 cycles of neoadjuvant immunotherapy combined with chemotherapy in patients with esophageal squamous cell carcinoma

**DOI:** 10.7150/jca.102215

**Published:** 2025-01-01

**Authors:** Yi Gan, Ting Bao, Zhiwei Tang, Chao Cheng, Haoshuai Zhu

**Affiliations:** 1 Department of Thoracic Surgery, the First Affiliated Hospital, Sun Yat-sen University, Guangzhou, China.; 2 Department of Thoracic Surgery, Hunan Cancer Hospital/The Affiliated Cancer Hospital of Xiangya School of Medicine, Central South University, Changsha, China.

**Keywords:** esophageal squamous cell carcinoma, immunotherapy combined with chemotherapy, efficacy, safety

## Abstract

**Background:** Neoadjuvant immunotherapy combined with chemotherapy has a substantial impact on locally advanced esophageal squamous cell carcinoma (LA-ESCC), but the optimal number of treatment cycles is still controversial.

**Method:** Patients who received 2 or 3 cycles of neoadjuvant immunotherapy combined with chemotherapy followed by esophagectomy to treat LA-ESCC were included. We compared the responses to neoadjuvant therapy, surgical outcomes, perioperative complications, and treatment-related adverse reactions in the two patient groups.

**Results:** A total of 100 patients were included in the study. The pathologic complete response (pCR) rate in patients who received 2 cycles was 18/56 (32.14%), and the pCR rate in patients who received 3 cycles was 14/44 (31.82%) (P=0.97). There was no significant difference in the perioperative parameters, postoperative complications or treatment-related adverse reactions between the two groups (P>0.05). After the third cycle, some patients experienced further relief, with a significant decrease in the NLR (P=0.0.4).

**Conclusion:** In LA-ESCC, the efficacy of both 2 cycles and 3 cycles of neoadjuvant immunotherapy combined with chemotherapy is comparable, with the same tolerance and feasibility. Further evaluation of the inflammation indicator NLR can help identify patients who would benefit from an additional third cycle of neoadjuvant therapy.

## Introduction

Esophageal cancer, mainly the squamous cell carcinoma subtype, is the fourth most significant cause of cancer-related deaths in China, and poses a severe threat to public health 1. Radical surgical resection remains the main treatment option for patients suffering from locally advanced esophageal squamous cell carcinoma (LA-ESCC). However, simple surgical interventions result in suboptimal outcomes, and high postoperative recurrence and metastasis rates strongly affect patient survival 2. In recent years, preoperative neoadjuvant immunotherapy combined with chemotherapy has been shown to have significant antitumor effects on LA-ESCC 3, 4. Based on the excellent results from the NICE study and several similar studies, the 2022 Chinese Society of Clinical Oncology (CSCO) guidelines have included the use of neoadjuvant immunotherapy combined with chemotherapy to treat LA-ESCC 5.

Currently, the number of cycles of neoadjuvant immunotherapy combined with chemotherapy that are administered to treat LA-ESCC varies among different centers, typically ranging from 2 to 3 cycles; this uncertainty arises from the absence of a standardized research protocol and a consistent method for evaluating efficacy 3, 4, 6, 7. Therefore, in this study, we conducted a comparative evaluation of the short-term efficacy and safety of 2 cycles and 3 cycles of neoadjuvant immunotherapy combined with chemotherapy in patients with LA-ESCC. All the patients were participants in clinical trials and received the same treatment regimen. The objective of this research was to determine the optimal number of neoadjuvant cycles required before surgery for patients with LA-ESCC, offering valuable insights for clinicians.

## Materials and Methods

### Patients and study design

This study included patients diagnosed with LA-ESCC who received 2 or 3 cycles of neoadjuvant immunotherapy combined with chemotherapy from January 2020 to April 2022 at the First Affiliated Hospital, Sun Yat-sen University. Patients were enrolled from the clinical trials ChiCTR2000028900 and ChiCTR1800019823. This study aimed to collect the clinical characteristics of the patients, details of the neoadjuvant treatments, surgical information, and postoperative pathology information for analysis. This study was conducted in accordance with the fundamental principles of the Declaration of Helsinki and ethical approval was obtained from the Clinical Research and Animal Experimentation Ethics Committee of the First Affiliated Hospital, Sun Yat-sen University.

The primary inclusion criteria were as follows: 1. Aged 18-75 years. 2.Pathologist-confirmed biopsy pathology for clinical TNM stage II-III locally advanced esophageal cancer patients 8. 2. Neoadjuvant treatment with the immunotherapy combined with chemotherapy. 3. Performance status (PS) score of 0 or 1. 4. Imaging examination to determine staging and exclude distant metastasis. 5. Availability of complete clinical and pathological data. 6. No history of other malignancies prior to treatment and no prior antitumor therapy. Exclusion criteria were as follows: 1. Pathologist-confirmed mixed adenosquamous carcinoma or other non-squamous carcinoma types. 2. Neoadjuvant treatments other than the combination of the PD-1 inhibitor camrelizumab with carboplatin and nab-paclitaxel. 3. No surgical treatment. 4. Fewer than 2 treatment cycles or more than 3 treatment cycles.

### Neoadjuvant therapy and surgical procedure

All the patients who met the inclusion and exclusion criteria were administered treatment once every three weeks. Camrelizumab at a dose of 200 mg was given on Day 1, nab-paclitaxel at a dose of 260 mg/m2 was given on Day 1, and carboplatin with an area under the curve (AUC) of 5, at a rate of 5 mg/mL/min was given on Day 1. Patients with LA-ESCC undergo preoperative assessment after completing 2-3 cycles of neoadjuvant therapy to evaluate the potential for surgery, and surgery is scheduled 21-42 days after the last treatment cycle. The primary tumor and lymph nodes were excised following the standard protocol for Minimally invasive esophagectomy (MIE), and the surgical method was three-incision esophagectomy 9.

### Efficacy and safety evaluation

The primary endpoint of the study was the pathologic complete response (pCR) rate. The secondary study endpoints include major pathologic response (MPR), R0 resection, immune-related toxicity, and perioperative safety. Pathological response was assessed according to the percentage of residual viable tumor after the primary tumor resection. pCR is characterized by the absence of viable tumor cells in the resected cancer specimen. mPR is defined by the presence of no more than 10% viable tumor cells, while pathological partial response (PR) is identified when viable tumor cells constitute more than 10% but no more than 50%. Pathological stable disease (SD) refers to cases with over 50% viable tumor cells 10-12.

Enhanced CT and/or positron emission tomography PET-CT and/or endoscopic ultrasound of the neck, chest, and upper abdomen were conducted before neoadjuvant therapy and after 2 or 3 cycles of neoadjuvant therapy. Radiological response was evaluated by two senior radiologists according to Response Evaluation Criteria in Solid Tumors (RECIST) version 1.1 as complete response (CR), partial response (PR), stable disease (SD), or progressive disease (PD) 13.

The patient's general condition after each neoadjuvant therapy were recorded, and the American Common Terminology Criteria for Adverse Events (CTCAE) version 5.0 was used to evaluate adverse reactions caused by neoadjuvant therapy. Additionally, postoperative complications were graded on the basis of the Clavien‒Dindo, and grade ≥3 complications were categorized major complications 14.

### Inflammatory indicator analysis

Routine blood data were collected before each neoadjuvant treatment, and white blood cell, lymphocyte, neutrophil, monocyte, and platelet counts were recorded. The neutrophil-to-lymphocyte ratio (NLR) is defined as the ratio of neutrophils to lymphocytes, the PLR is defined as the ratio of platelets to lymphocytes, and the lymphocyte-to-monocyte ratio (LMR) is defined as the ratio of lymphocytes to monocytes. The objective of this study was to compare the changes in the NLR, LMR, and PLR before treatment and after the second cycle of neoadjuvant treatment.

### Statistical analysis

Statistical analysis was performed with SPSS 23.0 (IBM Corp.). Normally distributed continuous variables are presented as the means ± standard deviations, whereas nonnormally distributed continuous variables are presented as medians and interquartile ranges. The t test was used to compare independent samples of normally distributed continuous variables, and the Mann‒Whitney U test was used to compare nonnormally distributed continuous variables. Comparisons of categorical variables between groups were made with Fisher's exact test or the Pearson χ2 test. Univariate logistic regression analysis was used to evaluate the influence of certain clinical variables on the pathological response. P<0.05 was considered to indicate a statistically significant difference.

## Results

### Patient characteristics

**Figure 1:**
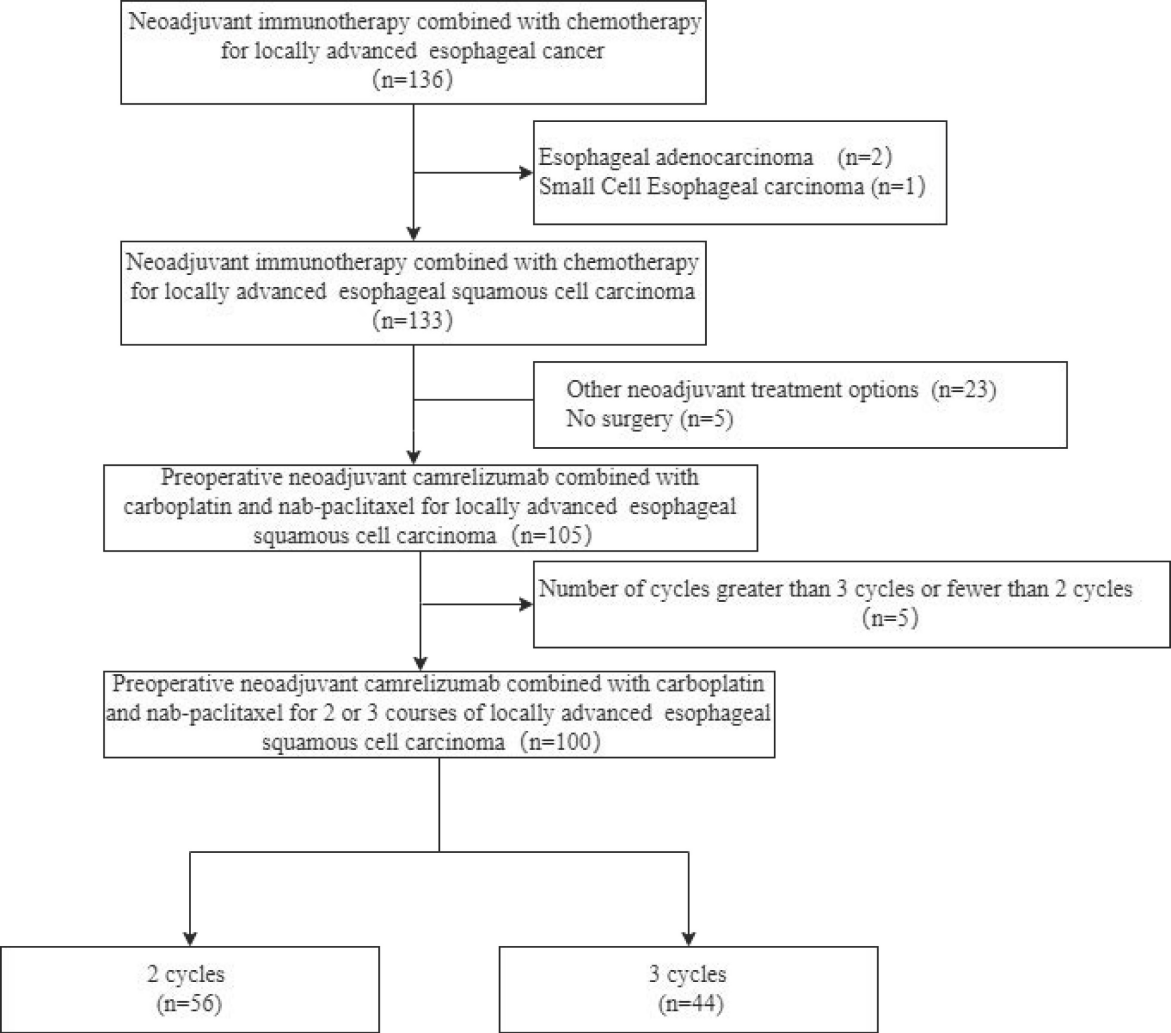
Study flow chart

From January 2020 to April 2022, a total of 136 patients were diagnosed with esophageal cancer Each of these patients underwent preoperative neoadjuvant immunotherapy combined with chemotherapy. The flow chart of this study is shown in Figure 1. Among these patients, a total of 100 patients were included in the research, including 56 patients who received 2 cycles and 44 patients who received 3 cycles of treatment. There were no significant differences in baseline characteristics including age, sex, PS score, and clinical TNM stage between the 2-cycle group and the 3-cycle group. All the clinical characteristics are shown in Table 1.

### Radiological response and pathological response

**Figure 2:**
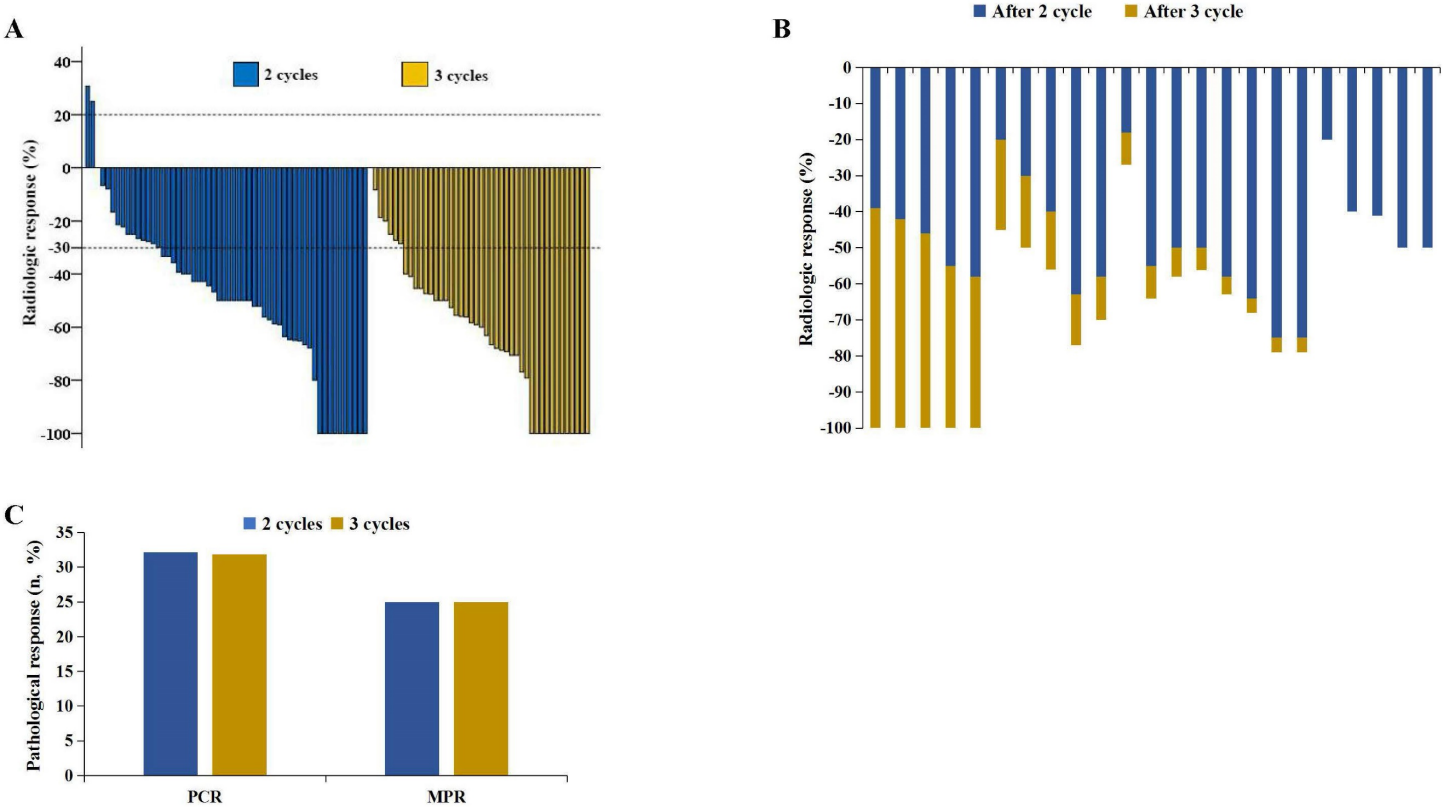
Radiological response and pathological response. A. Radiological responses in 100 patients. B. 23 patients experienced radiological changes during the second and third treatment cycles. C. Pathological responses.

**Figure 3:**
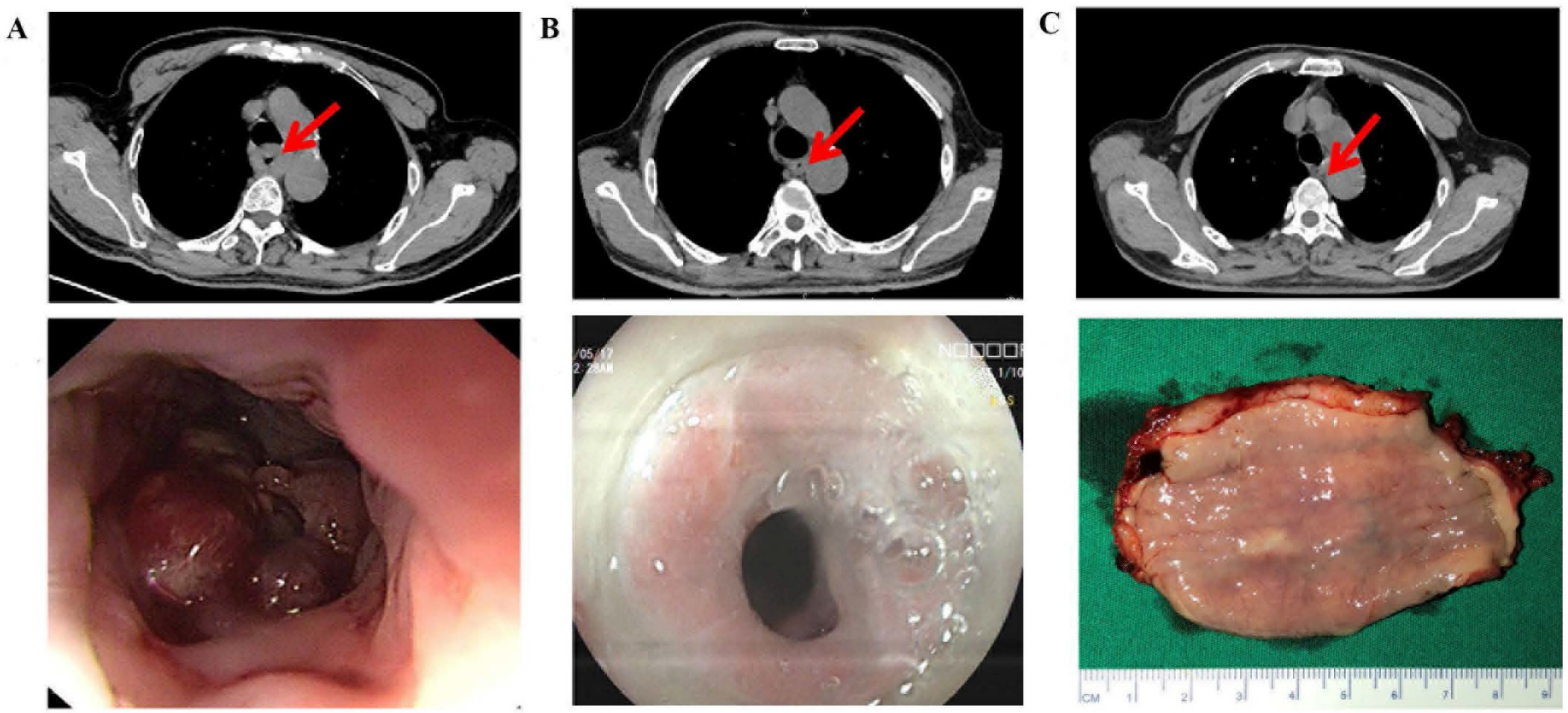
Radiographic response leading to pCR after three cycles of neoadjuvant therapy (top: cross section, bottom: gross view). A: Before treatment. B: tumor diameter regressed by 50% after the second. C: Tumor disappeared after the third cycle.

According to RECIST 1.1, 10 patients achieved CR after 2 cycles, whereas 11 patients achieved CR after 3 cycles (17.86% vs. 25.00%, P=0.38) (Figure 2A) (Table 2). Among the 100 patients who underwent surgery, 18 patients achieved pCR after 2 cycles, and 14 achieved pCR after 3 cycles (32.14% vs. 31.82%, P=0.97). Additionally, 14 patients achieved MPR after 2 cycles, and 11 achieved MPR after 3 cycles (25.00% vs. 25.00%, P=0.99) (Figure 2C). Pathological characteristics, including TNM downstaging and pathological response, were not significantly different between the two groups (P>0.05) (Table 2). Among the patients who completed 3 cycles of neoadjuvant therapy, 23 patients had pretreatment, post-2-cycle neoadjuvant treatment, and post-3-cycle neoadjuvant treatment radiological data. Among them, 5 patients further improved to CR after the third cycle of neoadjuvant treatment (Figure 2B) (Figure 3).

### Surgical outcomes

All the patients achieved R0 surgical resection. MIE was performed on 54 patients in the 2-cycle group and 44 patients in the 3-cycle group, with rates of 96.43% and 100.00%, respectively (P=0.50). There were no significant differences in the perioperative parameters, including surgical approach, surgical and postoperative conditions, or the number of patients who returned to the ICU between the two groups (P>0.05) (Table 2). Ninety-day mortality after surgery occurred in only one patient in the 3-cycle group, because of postoperative aspiration. Among the perioperative complications, the most common postoperative complication was anastomotic leakage (16.07% vs. 13.63%, P=0.79). The common major postoperative complications were dysphagia (3.57% vs. 4.55%, P=0.63) and intestinal obstruction (1.79% vs. 6.82%, P=0.32). Although the incidence of major postoperative complications after 3 cycles was slightly greater, but there was no significant difference in the incidence of major complications between the two groups (7.14% vs. 15.91%, P=0.21) (Table 2).

### Treatment-related adverse reactions

**Figure 4:**
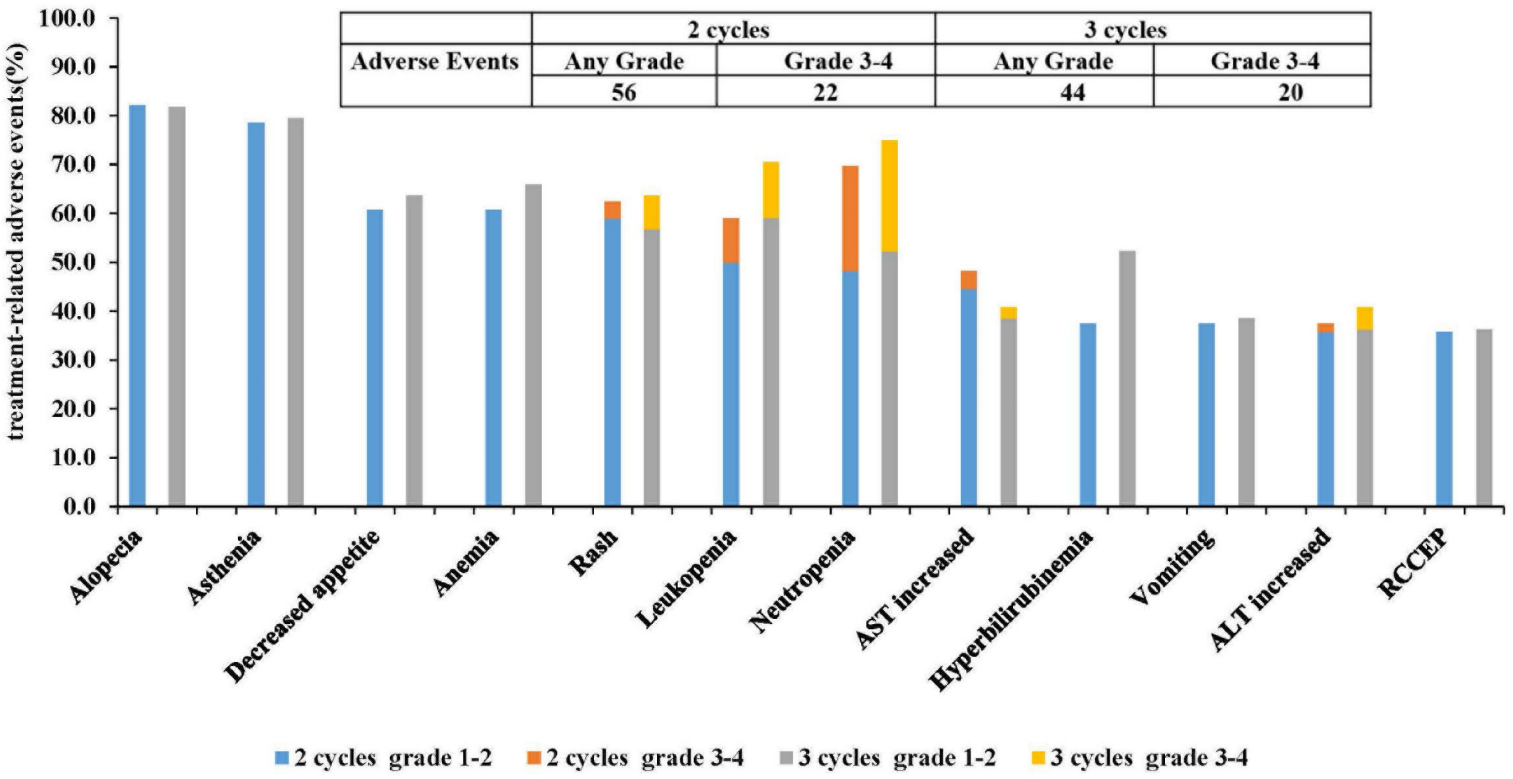
Treatment-related adverse events (The 12 most common adverse reactions) AST, aspartate aminotransferase ALT, alanine aminotransferase; RCCEP, reactive cutaneous capillary endothelial proliferation.

Treatment-related adverse reactions (TRAEs) occurred in all the patients. The most common TRAEs were alopecia (82.14% vs. 81.82%, P=0.97) and asthenia (78.57% vs. 79.55%, P=0.91). In 2 cycles and 3 cycles, there were 22 and 20 cases of grade 3-4 TRAEs, respectively (39.29% vs. 45.45%, P=0.54). The primary grade 3-4 TRAEs included neutropenia (21.43% vs. 22.73%, P=0.88) and leukopenia (8.93% vs. 11.36%, P=0.75). There was no statistically significant difference in the incidence of immunotherapy-related reactive cutaneous capillary endothelial proliferation (RCCEP) between two groups (35.71% vs. 36.36%, P=0.95). None of the TRAEs reported during neoadjuvant therapy resulted in treatment interruption, dose reduction, or surgical delay. Furthermore, there were no treatment-related deaths (Figure 4) (Supplemental Table 1).

### Univariate logistic regression analysis of risk factors for pathological response

In this study, we conducted univariate logistic regression analysis to evaluate whether baseline information and the number of previous neoadjuvant treatments were risk factors for pCR and MPR events, and the results identified no significant risk factors (Table 3).

### Inflammatory indicator analysis results

Patients with pretreatment and post-2-cycle neoadjuvant treatment radiological data were classified into two groups on the basis of the median percentage of further tumor diameter reduction after the third cycle of neoadjuvant treatment; 12 patients were included in the response group and 11 patients were included in the nonresponse group. The results revealed that there was no significant difference in the baseline NLR, PLR or LMR data before treatment between the two groups. We found that compared with the baseline data, after the second cycle of neoadjuvant immunotherapy combined with chemotherapy, the NLR (P=0.04) in the response group decreased significantly, whereas the changes in the PLR (P=0.24) and LMR(P=0.57) were not significant. In the nonresponsive group, there was no significant difference in the NLR P=0.84), PLR(P=0.26), or LMR(P=0.96) (Figure 4) (Supplemental Figure 1).

## Discussion

Esophageal squamous cell carcinoma is characterized by its widespread occurrence and high mortality rate 15. Preoperative neoadjuvant therapy is being explored as a potential approach to extend postoperative survival in patients undergoing radical resection 16, 17. The combination of the PD-1 inhibitor camrelizumab with chemotherapy has the potential to improve the survival time of patients with LA-ESCC while ensuring safety 18. However, there is controversy in the literature regarding about the optimal number of cycles for neoadjuvant immunotherapy combined with chemotherapy. Our study revealed that for patients with LA -ESCC, 2 cycles and 3 cycles of neoadjuvant therapy combined with chemotherapy are equally effective and have the same tolerability and feasibility. To the best of our knowledge, this study represents the first attempt to compare the efficacy and safety of different numbers of cycles of neoadjuvant immunotherapy combined with chemotherapy in LA-ESCC patients.

In our study, all the patients achieved R0 surgical resection. The rates of pCR among patients who received 2 cycles and 3 cycles of neoadjuvant therapy were similar, consistent with findings from existing studies on neoadjuvant camrelizumab combined with chemotherapy in locally advanced esophageal squamous cell carcinoma 19-22. Previous studies demonstrated that the pCR rates of 2 and 3 cycles of neoadjuvant chemotherapy were comparable (9.1% vs. 15.3%, P = 0.212). In this study, despite improved pathological outcomes in both groups after the addition of immunotherapy, no significant difference in pathological response was observed between the groups (32.14% vs. 31.82%, P = 0.97). Additionally, there was no significant correlation between the two groups in terms of pathological characteristics, including pCR, MPR, T downstaging, N downstaging, TNM downstaging, or other pathological responses. Our results indicate that 2 and 3 cycles of neoadjuvant therapy are equally feasible. In this study, there was no significant correlation between the two groups in terms of pathological characteristics, including pCR, MPR, T downstaging, N downstaging, TNM downstaging, and other pathological response. Our results indicate that 2 and 3 cycles of neoadjuvant therapy are equally feasible.

Immunotherapy may trigger severe tissue reactions, potentially leading to dense fibrosis in the surgical area 23, 24. However, in our study, additional cycle of neoadjuvant therapy did not increase the degree of difficulty of surgery. Perioperative parameters, such as operative time, chosen surgical approach and blood loss, were not significantly different between the two groups. In terms of postoperative complications, there was no significant difference between the two groups after 2 cycles and 3 cycles. The most common postoperative complication observed in this study was anastomotic leakage, with probabilities of 16.07% in the 2-cycle group and 13.63% in the 3-cycle group, P=0.79. Our results indicate that additional cycle of neoadjuvant immunotherapy combined with chemotherapy has no significant impact on postoperative complications.

In both the 2-cycle and 3-cycle groups, neoadjuvant immunotherapy combined with chemotherapy had an acceptable safety profile in patients with LA-ESCC. Although all the patients experienced treatment-related adverse effects, these events were generally mild in both groups. Grade 3-4 hematological toxicity-related adverse reactions occurred in 39.29% of patients in the 2-cycle group, compared with 45.456% in the 3-cycle group, with no significant difference between the two groups. This result is comparable to the 42.2% reported in the TD-NICE clinical trial, where patients received 3-cycle of neoadjuvant chemotherapy combined with immunotherapy. Our results demonstrated that adding an additional cycle of neoadjuvant therapy did not lead to an increase in treatment-related TRAEs. Importantly, these adverse reactions during neoadjuvant treatment did not necessitate treatment interruption, dose reduction, or surgical delays for either group of patients.

A meta-analysis of currently available data showed no statistical correlation between the number of neoadjuvant treatment cycles and the MPR rate, pCR rate, TRAE rate, or surgical complication rate in neoadjuvant chemoimmunotherapy, which is consistent with our findings 25. However, due to the small sample size and the varying medication regimens from different institutions, this conclusion still needs to be validated by clinical studies with larger sample sizes.

The introduction of immunotherapy has complicated the assessment of clinical research efficacy, as some lesions showed increased tumor volume or new lesions due to lymphocyte infiltration, edema, and necrosis after neoadjuvant therapy. These changes were classified as PD in the radiological evaluation, although they did not indicate actual tumor cell proliferation. To minimize research inaccuracies, this article focuses primarily on pathological outcomes as the main endpoint. In many clinical trials, pCR and MPR are recognized as surrogate markers for overall survival and progression-free survival 26, 27. We conducted an assessment to determine whether various clinical parameters, serve as risk factors for pCR/MPR. The results revealed that no risk indicators significantly influenced the pCR/MPR outcome.

Inflammatory indicators in blood, such as the NLR, PLR, and LMR, are associated with tumor development and progression 28, 29. In our study, we observed that some patients experienced further relief after an additional third cycle. Analysis of laboratory data revealed that patients who achieved additional relief during the third cycle of neoadjuvant therapy exhibited a significant reduction in the NLR after the second cycle of neoadjuvant therapy. The NLR is now considered a biomarker for predicting overall survival and the effect of anti-PD-1/PD-L1 treatment in patients with different types of tumors, including esophageal squamous cell carcinoma 30, 31. This decrease in the NLR was associated with an increase in lymphocyte count and a decrease in the neutrophil count. Studies have demonstrated that lymphocytes play pivotal roles in inhibiting tumor growth and improving cancer patient survival by producing cytokines such as IFN-γ and TNF-α 32. Neutrophils play a significant role in tumor progression and metastasis by releasing factors that inhibit the activation and antitumor effects of CD8+ T cells, leading to abnormal extracellular matrix remodeling and angiogenesis regulation 33, 34.

Our study presents several advantages. First, the majority of patients in this study were selected from the clinical trials ChiCTR2000028900 and ChiCTR1800019823. Second, all the patients included in this study received the same treatment regimen (neoadjuvant camrelizumab combined with carboplatin and nab-paclitaxel). However, this study has several limitations. First, this was a single-center retrospective study, and the number of patients in both the 2-cycle and 3-cycle groups was relatively small, which may introduce experimental bias. In the future, these results should be verified in large-scale, prospective, multicenter studies. Second, given the relatively short follow-up period, the study is currently unable to provide comparisons with 5-year survival-related data. We plan to include more patients and conduct long-term follow-ups for further investigation.

## Supplementary Material

Supplementary figure and tables.

## Figures and Tables

**Figure 5:**
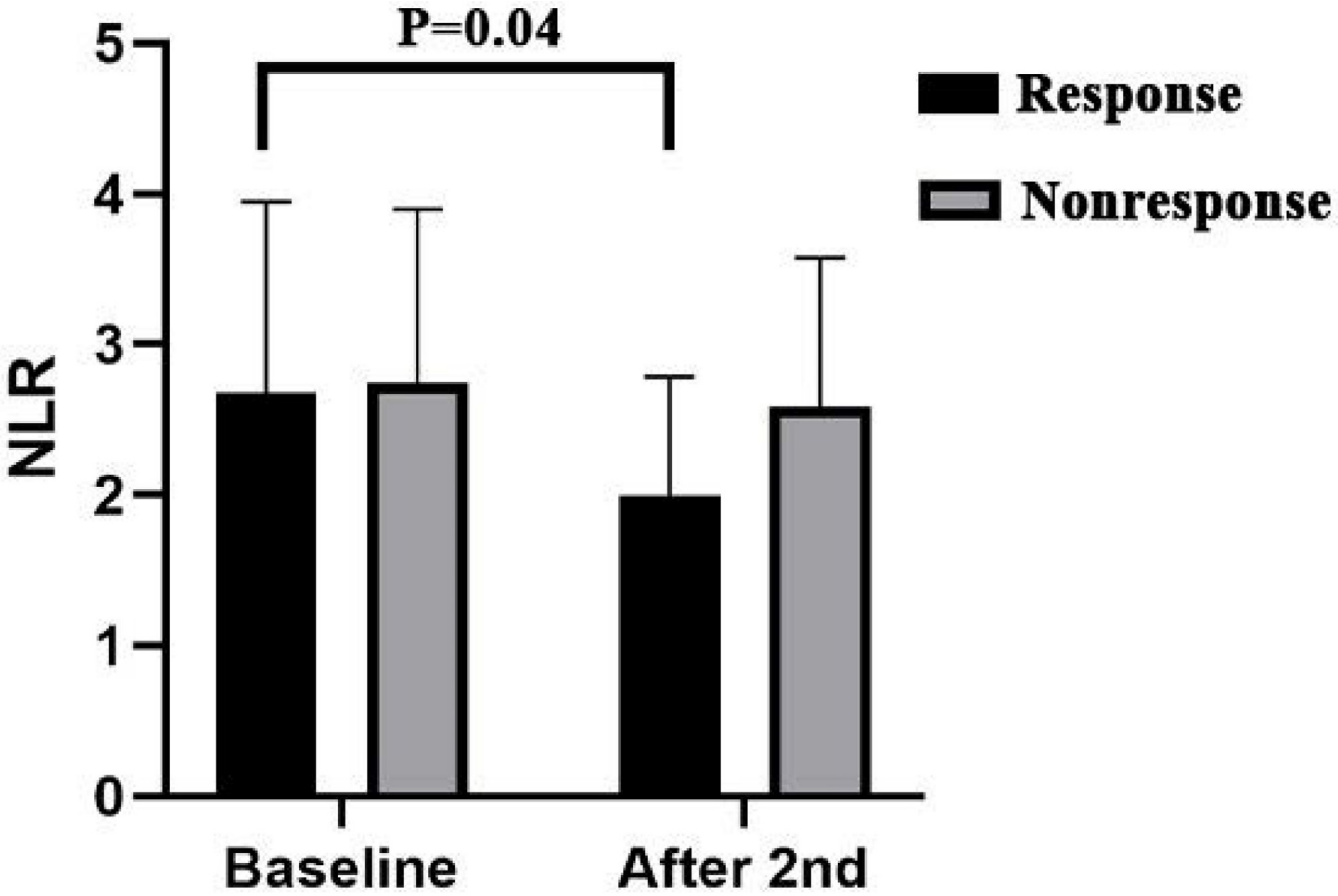
Inflammatory indicators analysis results. NLR, Neutrophil-to-Lymphocyte Ratio.

**Table 1:** Demographic and tumor characteristics of patients

Characteristic	2 cycles	3 cycles	P-value
n	56 (100.00)	44 (100.00)	
Age (years)^*^	61.91±8.40	59.98±7.70	0.23
Sex, n (%)			0.31
Male	44 (78.57)	38 (86.36)	
Female	12 (21.43)	6 (13.64)	
Smoking status, n (%)			0.42
Never	21 (37.50)	20 (45.45)	
Former or current	35 (62.50)	24 (54.56)	
Alcohol consumption, n (%)			0.42
Never	30 (53.57)	25 (56.82)	
Former or current	26 (46.43)	19 (43.18)	
Location, n (%)			0.46
Upper segment	8 (14.29)	8 (18.18)	
Middle segment	26 (46.43)	15 (34.09)	
Lower segment	22 (39.28)	21 (47.73)	
cT, n (%)			0.35
2	9 (16.07)	3 (6.82)	
3	44 (78.57)	37 (84.09)	
4	3 (5.63)	4 (9.09)	
cN, n (%)			0.45
0	22 (39.29)	16 (36.36)	
1	27 (48.21)	17 (38.64)	
2	5 (8.93)	8 (18.18)	
3	2 (3.57)	3 (6.82%)	
cStage, n (%)			0.15
2	27 (51.11)	13 (32.35)	
3	24 (42.22)	24 (52.94)	
4	5 (6.67)	7 (14.71)	
PS score, n (%)			0.63
0	53 (94.64)	43 (97.73)	
1	3 (5.36)	1 (2.27)	

*, mean ± standard deviation; **, median ± interquartile range; cT, clinical Tumor; cN, clinical Node; cStage, clinical Stage; PS score, Performance Status score.

**Table 2:** Surgical and pathological outcomes

Characteristics	2 cycles	3 cycles	P-value
n	56 (100.00)	44 (100.00)	
Successful R0 resection, n (%)	56 (100.00)	44 (100.00)	
Surgical approach, n (%)			
MIE, n (%)	54 (96.43)	44 (100.00)	0.50
OE, n (%)	2 (3.57)	0 (0.00)	
Radiological response, n (%)			
CR	10 (17.86)	11 (25.00)	0.38
PR	33 (58.93)	26 (59.09)	0.99
SD	11 (19.64)	7 (15.91)	0.66
PD	2 (3.57)	0 (0.00)	0.59
Pathological response, n (%)			
PCR	18 (32.14)	14 (31.82)	0.97
MPR	14 (25.00)	11 (25.00)	0.99
PR	12 (21.43)	15 (34.09)	0.17
SD	12 (21.43)	4 (9.09)	0.11
Downstaging of T stage, n (%)	43 (76.79)	39 (88.64)	0.13
Downstaging of N stage, n (%)	24 (42.86)	22 (50.00)	0.48
Downstaging of TNM stage, n (%)	38 (67.86)	36 (81.82)	0.11
Blood loss (mL)^**^	100.00 (100.00-150.00)	100.00 (100.00-200.00)	0.41
Cumulative operative time (min)^**^	273.00 (252.75-290.00)	274.42 (251.25-299.75)	0.42
Number of resected lymph nodes^ **^	28.13 (21.00-34.00)	29.27 (22.00-36.75)	0.54
Number of resected lymph node stations^ *^	9.30 (7.00-11.00)	9.23 (8.00-11.00)	0.81
Transition thoracotomy, n (%)	0 (0.00)	0 (0.00)	
Chest drainage duration (days)^ **^	5.00 (4.00-6.00)	5.00 (4.00-6.00)	0.72
Chest drainage volume (ml)^ **^	1585.00 (1205.00-1900.00)	1640.00 (1285.00-2275.00)	0.68
ICU stay, n (%)	3 (5.36)	6 (13.64)	0.18
perioperative complications			
Clavien-Dindo grade I-II, n (%)	14 (25.00)	14 (31.82)	0.57
Postoperative hoarseness	1 (1.79)	1 (2.27)	0.99
Anastomotic leakage	9 (16.07)	6 (13.63)	0.79
Cardiac event	2 (3.57)	3 (6.82)	0.65
Pulmonary infection	2 (3.57)	4 (9.09)	0.40
Clavien-Dindo grade III-IV, n (%)	4 (7.14)	7 (15.91)	0.21
Dysphagia after surgery	2 (3.57)	2 (4.55)	0.63
Pleural effusion	0 (0.00)	1 (2.27)	0.44
Intestinal obstruction	1 (1.79)	3 (6.82)	0.32
Ascites	1 (1.79)	1 (2.27)	0.99
90-day mortality	0 (0.00)	1 (2.27)	0.44
			

*, mean ± standard deviation; **, median ± interquartile range. MIE, minimally invasive esophagectomy; OE, open esophagectomy; PCR, pathological complete response; MPR, major pathological response; PR, partial response; SD, stable disease; TNM, Tumor Node Metastasis.

**Table 3:** Univariate logistic regressive analysis for pathological response

Characteristic	CR/MPR (n=57)	Non-CR/MPR (n=43)	P-value
n	57 (100.00)	43 (100.00)	
Sex, n (%)			0.92
Male	46 (80.70)	36 (83.72)	
Female	11 (19.30)	7 (16.28)	
Age (years)* *	60.00 (53.50-67.00)	63.00 (57.00-68.00)	0.06
Smoking status, n (%)			0.34
Never	23 (40.35)	18 (41.86)	
Former or current	34 (59.65)	25 (58.14)	
Alcohol consumption, n (%)			0.52
Never	33 (57.89)	22 (51.16)	
Former or current	24 (42.11)	21 (48.84)	
Location, n (%)			0.18
Upper segment	11 (19.30)	5 (11.62)	
Middle segment	22 (38.60)	19 (44.19)	
Lower segment	24 (42.10)	19 (44.19)	
cT, n (%)			0.34
2	6 (10.53)	6 (13.95)	
3	48 (84.21)	33 (76.74)	
4	3 (5.26)	4 (9.31)	
cN, n (%)			0.09
0	22 (38.60)	16 (37.21)	
1	24 (42.11)	20 (46.11)	
2	9 (15.79)	4 (9.30)	
3	2 (3.50)	3 (7.38)	
cStage, n (%)			0.08
2	24 (42.11)	16 (37.21)	
3	28 (49.12)	20 (46.51)	
4	5 (8.77)	7 (16.28)	
Performance score, n (%)			0.57
0	55 (96.49)	41 (95.35)	
1	2 (3.51)	2 (4.65)	
Cycles, n (%)			0.52
2	32 (56.14)	24 (55.81)	
3	25 (43.86)	19 (44.19)	

*, mean ± standard deviation; **, median ± interquartile range; PCR, pathological complete response; MPR, major pathological response; cT, clinical Tumor; cN, clinical Node; cStage, clinical Stage; PS score, Performance Status score.
