# Supplementary material for: Analysis of the short-term efficacy of 2 versus 3 cycles of neoadjuvant immunotherapy combined with chemotherapy in patients with esophageal squamous cell carcinoma
Source: J Cancer. 2025 Jan 1;16(1):279–87. doi: 10.7150/jca.102215 (PMC11660122; doi:10.7150/jca.102215)
Supplement: Supplementary file 1 — Supplementary figure and tables. [file jcav16p0279s1.pdf]

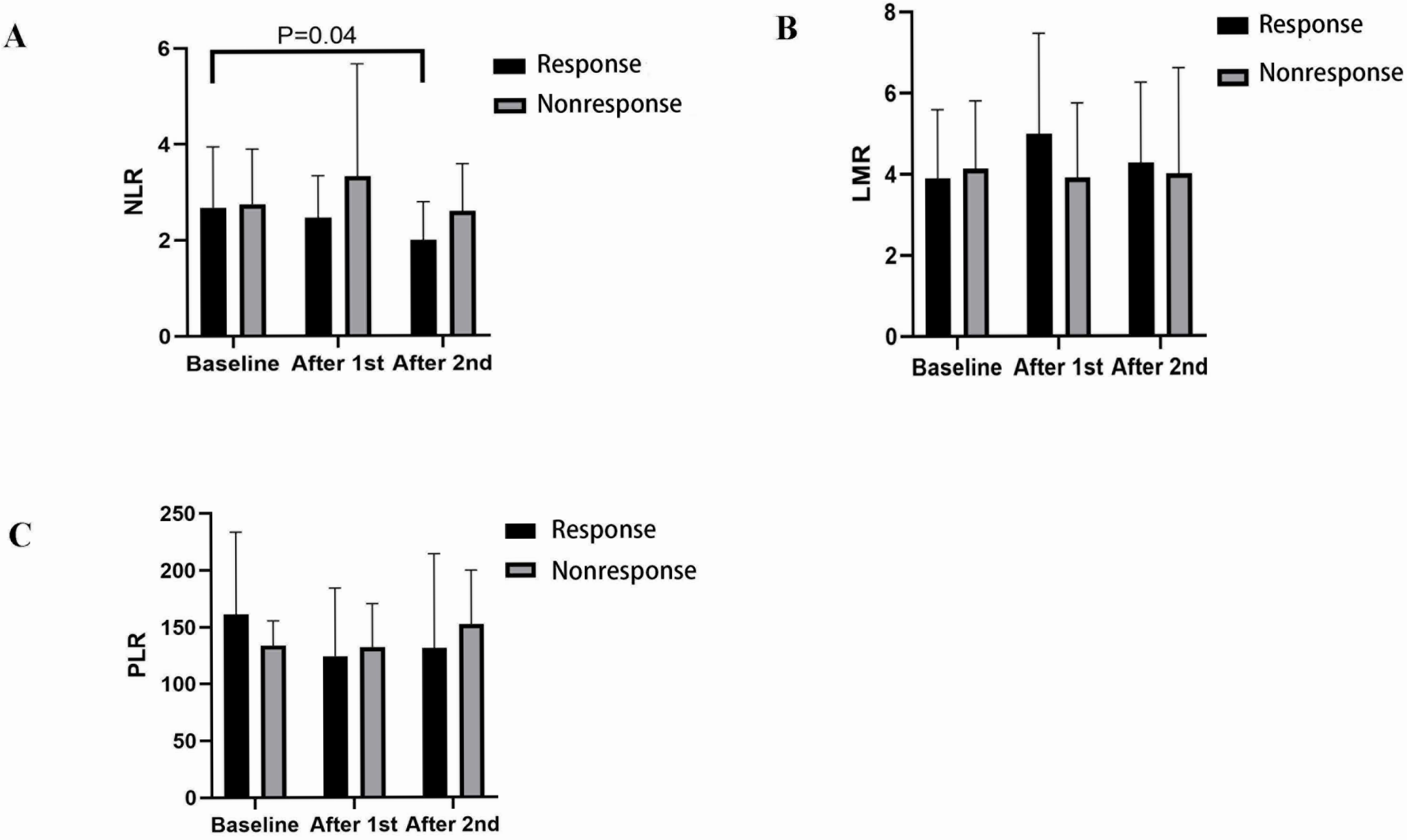

Supplemental Figure 1

Supplement Table 1 Treatment-related adverse events

Characteristic(n,100%)	2 cycles Any Grade	3 cycles Any Grade	P value	2 cycles Grade 3-4	3 cycles Grade 3-4	P value
n,100%	56 (100.00)	44(100.00)		22 (39.29)	20 (45.45)	0.54
Alopecia	46 (82.14)	36 (81.82)	0.97			
Asthenia	44 (78.57)	35 (79.55)	0.91			
Decreased appetite	34 (60.71)	28 (63.63)	0.77			
Anemia	34 (60.71)	29 (65.91)	0.59			
Rash	33 (58.93)	25 (56.82)	0.37	2 (3.57)	3 (6.82)	0.65
Leukopenia	28 (50.00)	26 (59.09)	0.37	5 (8.93)	5 (11.36)	0.75
Neutropenia	27 (48.21)	23 (52.27)	0.69	12 (21.43)	11 (22.73)	0.88
Aspartate aminotransferase increased	25 (44.64)	17 (38.63)	0.55	2 (3.57)	1 (2.27)	0.99
Hyperbilirubinemia	21(37.50)	23 (52.27)	0.14			
Vomiting	21 (37.50)	17 (38.63)	0.91			
Alanine aminotransferase increased	20 (35.71)	16 (36.36)	0.95	1 (1.79)	2 (4.55)	0.58
Reactive cutaneous capillary endothelial proliferation	20 (35.71)	16 (36.36)	0.95			
Thrombocytopenia	16 (28.57)	14 (31.82)	0.73			
Constipation	15 (26.79)	13 (29.55)	0.76			
Diarrhea	11 (19.64)	11 (22.73)	0.52			
Dyspnea	4 (7.14)	2 (4.55)	0.69			
Oral mucositis	4 (7.14)	2 (4.55)	0.69			
Nausea	3 (5.36)	4 (9.09)	0.70			
Hyperthyroidism	3 (5.36)	3 (6.82)	0.99			
Fever	1 (1.79)	2 (4.55)	0.58			
Peripheral sensory neuropathy	3 (5.36)	4 (9.09)	0.70			
Arthralgia	2 (3.57)	4 (9.09)	0.40			
